# Cisplatin induces stemness in ovarian cancer

**DOI:** 10.18632/oncotarget.8852

**Published:** 2016-04-20

**Authors:** Andrew Wiechert, Caner Saygin, Praveena S. Thiagarajan, Vinay S. Rao, James S. Hale, Nikhil Gupta, Masahiro Hitomi, Anil Belur Nagaraj, Analisa DiFeo, Justin D. Lathia, Ofer Reizes

**Affiliations:** ^1^ Department of Cellular and Molecular Medicine, Lerner Research Institute, Cleveland Clinic, Cleveland, OH 44195, USA; ^2^ Division of Gynecological Oncology, Women's Health Institute, Cleveland Clinic, Cleveland, OH 44195, USA; ^3^ Cleveland Clinic Lerner College of Medicine of Case Western Reserve University, Cleveland, OH 44195; ^4^ Department of Molecular Medicine, Cleveland Clinic Lerner College of Medicine at Case Western Reserve University, Cleveland, OH 44195, USA; ^5^ Department of Case Comprehensive Cancer Center, Cleveland, OH 44106, USA

**Keywords:** cancer stem cell, ovarian cancer, cisplatin, NANOG

## Abstract

The mainstay of treatment for ovarian cancer is platinum-based cytotoxic chemotherapy. However, therapeutic resistance and recurrence is a common eventuality for nearly all ovarian cancer patients, resulting in poor median survival. Recurrence is postulated to be driven by a population of self-renewing, therapeutically resistant cancer stem cells (CSCs). A current limitation in CSC studies is the inability to interrogate their dynamic changes in real time. Here we utilized a GFP reporter driven by the NANOG-promoter to enrich and track ovarian CSCs. Using this approach, we identified a population of cells with CSC properties including enhanced expression of stem cell transcription factors, self-renewal, and tumor initiation. We also observed elevations in CSC properties in cisplatin-resistant ovarian cancer cells as compared to cisplatin-naïve ovarian cancer cells. CD49f, a marker for CSCs in other solid tumors, enriched CSCs in cisplatin-resistant and -naïve cells. NANOG-GFP enriched CSCs (GFP+ cells) were more resistant to cisplatin as compared to GFP-negative cells. Moreover, upon cisplatin treatment, the GFP signal intensity and NANOG expression increased in GFP-negative cells, indicating that cisplatin was able to induce the CSC state. Taken together, we describe a reporter-based strategy that allows for determination of the CSC state in real time and can be used to detect the induction of the CSC state upon cisplatin treatment. As cisplatin may provide an inductive stress for the stem cell state, future efforts should focus on combining cytotoxic chemotherapy with a CSC targeted therapy for greater clinical utility.

## INTRODUCTION

Ovarian cancer is the most lethal gynecological malignancy in the United States [1]. Over the past three decades, advances in cytotoxic chemotherapy have allowed a subset of patients to survive for 3–5 years [1]. While initial treatment with taxane-platinum combination chemotherapy and debulking surgery allows eighty percent of patients to achieve clinical remission, the vast majority of these patients recur with a median time to recurrence of 12–24 months [2, 3]. The remaining twenty percent of patients fail initial treatment, with progression of disease either during or within the first six months following chemotherapy. Relapsed ovarian cancer is universally incurable and current standard of care is cytotoxic chemotherapy with symptomatic management [4]. Data from large genetic analyses, including The Cancer Genome Atlas, has demonstrated that epithelial ovarian cancer is a genetically heterogeneous disease, and has failed to identify targetable driver gene mutations for the majority of patients [5]. Thus, an alternative strategy must be employed to define signaling pathways which may be targeted to either enhance chemotherapeutic response by synthetic lethality, or render otherwise chemo-resistant cells susceptible to currently used drugs such as platinum or taxanes.

Ovarian cancer is marked by a high degree of cellular heterogeneity and contains a cancer stem cell (CSC) population that contributes to tumor growth and treatment resistance [6–9]. Several reports have demonstrated a subpopulation of CSCs in ovarian cancer, which constitutes a dynamic model with genetic mutations contributing to conversion of non-CSCs to CSCs, and vice versa [6–9]. CSCs are defined by their ability to self-renew and form tumors at a high frequency in immune-compromised mice [10]. Ovarian CSCs have been enriched both by selective cell surface expression (including CD44, CD24, CD133, CD117, CA125 cell surface markers) and enzymatic activity (via ALDH) and validated by functional analyses [8, 9, 11–15]. However, these enrichment methods are limited in their ability to assess the stem cell activity in real time, and do not provide a direct readout of how a given therapy or pathway inhibition can alter CSC maintenance.

A hallmark of CSCs is high expression of embryonic stem cell transcription factors that are essential of self-renewal. In particular, NANOG has been reported to be a master regulator of stem cell maintenance in both the normal and neoplastic context [16, 17]. NANOG is elevated in breast and prostate cancer and suggested to be an oncogene [18, 19]. Here we report the generation of a CSC reporter system based on NANOG promoter activity that provides a platform to monitor the CSC state in real time. This reporter system can reliably enrich tumor-initiating and cisplatin resistant CSCs out of cisplatin-naïve ovarian cancer cells. In parallel, we found that CD49f, a known CSC marker, can enrich for self-renewing CSCs in cisplatin-naïve and -resistant cell models. Finally, we found that cisplatin treatment induces CSC state based on our reporter system.

## RESULTS

CSCs have been enriched in solid tumors based on reporter system activity [20–24]. Leveraging our previous success in the enrichment of CSCs in triple-negative breast cancer using a NANOG promoter-driven green fluorescence protein (GFP) reporter system [25], we applied this to ovarian cancer cell lines.

### Development of ovarian cancer reporter system to select CSCs

**Figure 1:**
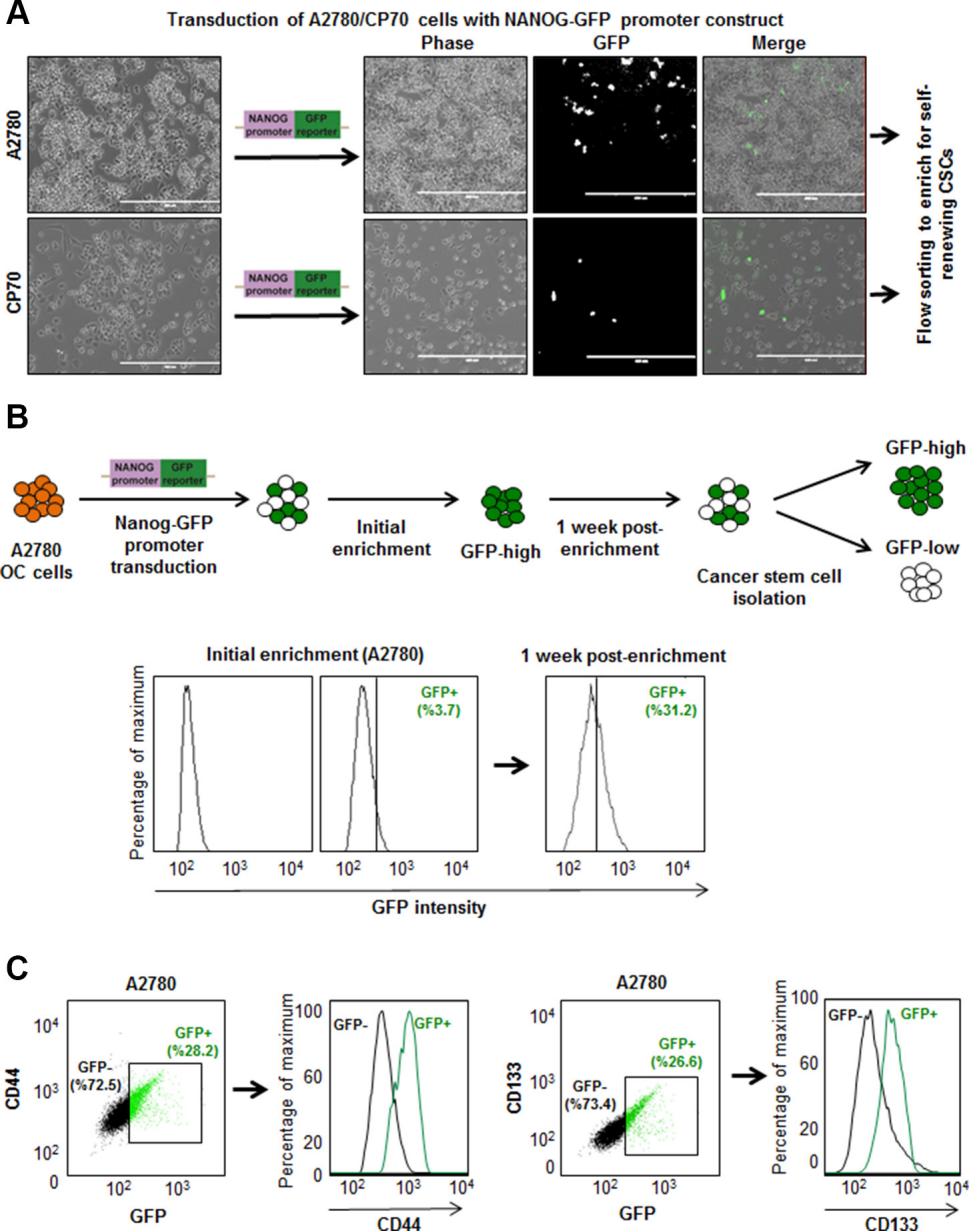
Development of ovarian cancer reporter system to enrich CSCs (**A**) Transduction of ovarian cancer cell lines with NANOG-GFP reporter and representative photomicrographs of cultured cells post transduction. (**B**) The workflow of generating stably transduced ovarian cancer cell lines. After initial enrichment and expansion of GFP+ cells, we observed the repopulation of original tumor heterogeneity at 1 week post-enrichment. (**C**) A2780 NANOG-GFP cells were stained with PE-CD44 and APC-CD133, and analyzed by flow cytometry. The green and black dots and histogram lines represent GFP+ and GFP–cells, respectively. Histograms demonstrate that GFP+ cells are enriched in CD44 and CD133 expression.

**Figure 2:**
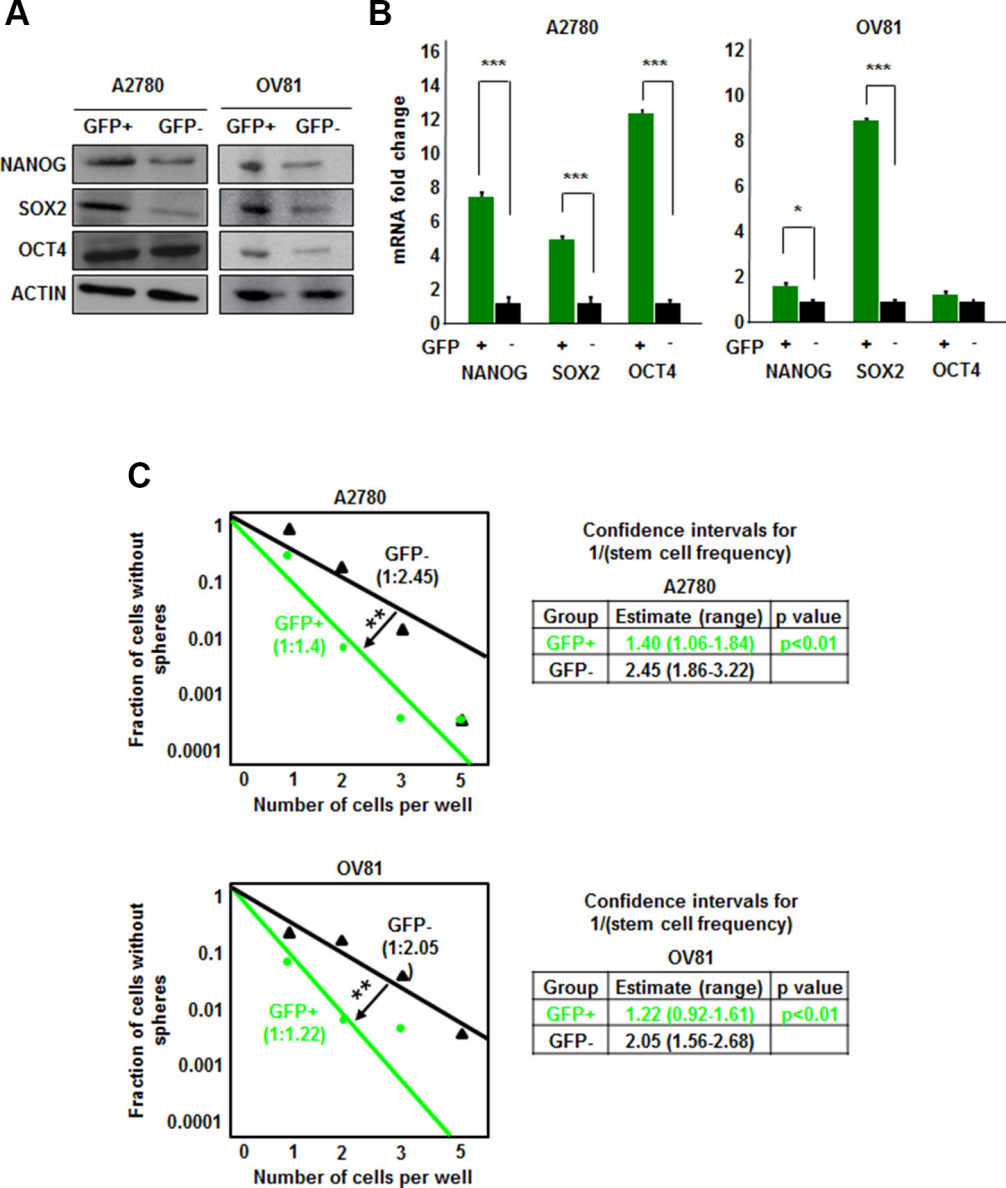
NANOG-GFP+ A2780 and OV81 cells are enriched for CSCs (**A**) Immunoblots of GFP–sorted A2780 and OV81 cells probed with antibodies to the stem cell transcription factors. A2780 GFP+ cells have higher expression of NANOG and SOX2 than GFP negative cells. Similarly, OV81 GFP+ cells have higher levels of NANOG, SOX2, and OCT4 proteins than GFP–cells. Actin was used as a loading control. (**B**) Quantitation of NANOG, SOX2, and OCT4 mRNAs in GFP-sorted A2780 and OV81 cells showed significantly higher expression levels in GFP+ cells compared to their GFP–counterparts. (**C**) Limiting dilution assays were performed by plating cells into 96-well plates with increasing cell numbers. GFP+ A2780 and OV81 cells had higher self-renewal capacity and stem cell frequencies as compared to their negative counterparts. Values represent mean +/− standard deviation, *p < 0.05, **p < 0.01, ***p < 0.001, as assessed by one-way-ANOVA.

We transduced isogenic cisplatin-naïve (A2780) and –resistant (CP70) ovarian cancer cell lines with our NANOG-GFP reporter system (Figure 1A, 1B). We also introduced the reporter into cisplatin-naïve high-grade serous ovarian cancer (HGSOC) patient-derived xenograft (PDX), OV81. After purification of GFP positive cells using flow cytometry, we initiated cell cultures that displayed heterogeneity and found A2780 GFP high cells also expressed higher levels of CSC markers including CD44, CD133, CD117, and CD24 (Figure 1C, Supplementary Figure 1). We found that both A2870 and OV81 GFP+ cells had higher expression of NANOG and SOX2 at protein and RNA levels (Figure 2A, 2B). GFP+ A2780 cells had 7.5, 5 and 12.6 fold higher levels of NANOG, SOX2 and OCT4 mRNA as compared to GFP- cells, respectively (Figure 2B). Similarly, GFP+ OV81 cells had 1.8 and 8.5 fold higher expression of NANOG and SOX2 mRNA as compared to their GFP- counterparts (Figure 2B). In CP70 cells, the difference in GFP+ and GFP- cells was not as pronounced (Supplementary Figure 2). To assess self-renewal, a key functional CSC hallmark, we performed limiting dilution sphere formation analysis that provides readout for self-renewal, proliferation, and survival. We found that A2870 GFP+ cells were significantly more self-renewing than their GFP- counterparts (stem cell frequencies were 1:1.4; confidence interval = 1:1.1–1:1.8, and 1:2.45; confidence interval = 1:1.9–1:3.2 in GFP+ vs GFP- cells, respectively) (Figure 2C). Similarly, GFP+ OV81 cells had significantly higher self-renewal capacity as compared to their GFP- counterparts (stem cell frequencies were 1:1.22 [confidence interval = 1:0.92–1:1.61], and 1:2.05 [confidence interval = 1:1.56–1:2.68] in GFP+ vs GFP- cells, respectively) (Figure 2C). The difference of reporter system fidelity between cisplatin-naïve and resistant lines could be due to the difference in baseline CSC transcription factor expression and self-renewal capacity. CP70 cells display elevated NANOG and OCT4 protein and RNA as compared with A2780 cells (Supplementary Figure 3A, 3B). We observed similar increase in NANOG and OCT4 levels in CP10 cell line, as compared to its isogenic origin OV81 cells (Supplementary Figure 3C, 3D). Additionally, CP70 cells possessed higher self-renewal as compared to A2780 cells (stem cell frequencies were 1:1.34 [confidence interval = 1:1–1:1.8], and 1:0.9 [confidence interval = 1:0.6–1:1.2] in A2780 vs CP70 cells, respectively) (Supplementary Figure 3E). Similarly, CP10 cells had higher self-renewal capacity as compared to OV81 cells (stem cell frequencies were 1:2.95 [confidence interval = 1:2.22–1:3.92], and 1:1.93 [confidence interval = 1:1.47–1:2.52] in OV81 vs CP10 cells, respectively) (Supplementary Figure 3E). These data demonstrate that our NANOG-GFP reporter system has the capacity to enrich for CSCs in cisplatin-naïve A2780 and OV81 cells but not in cisplatin-resistant CP70 cells.

### CSCs are also present in cisplatin-resistant cells

**Figure 3:**
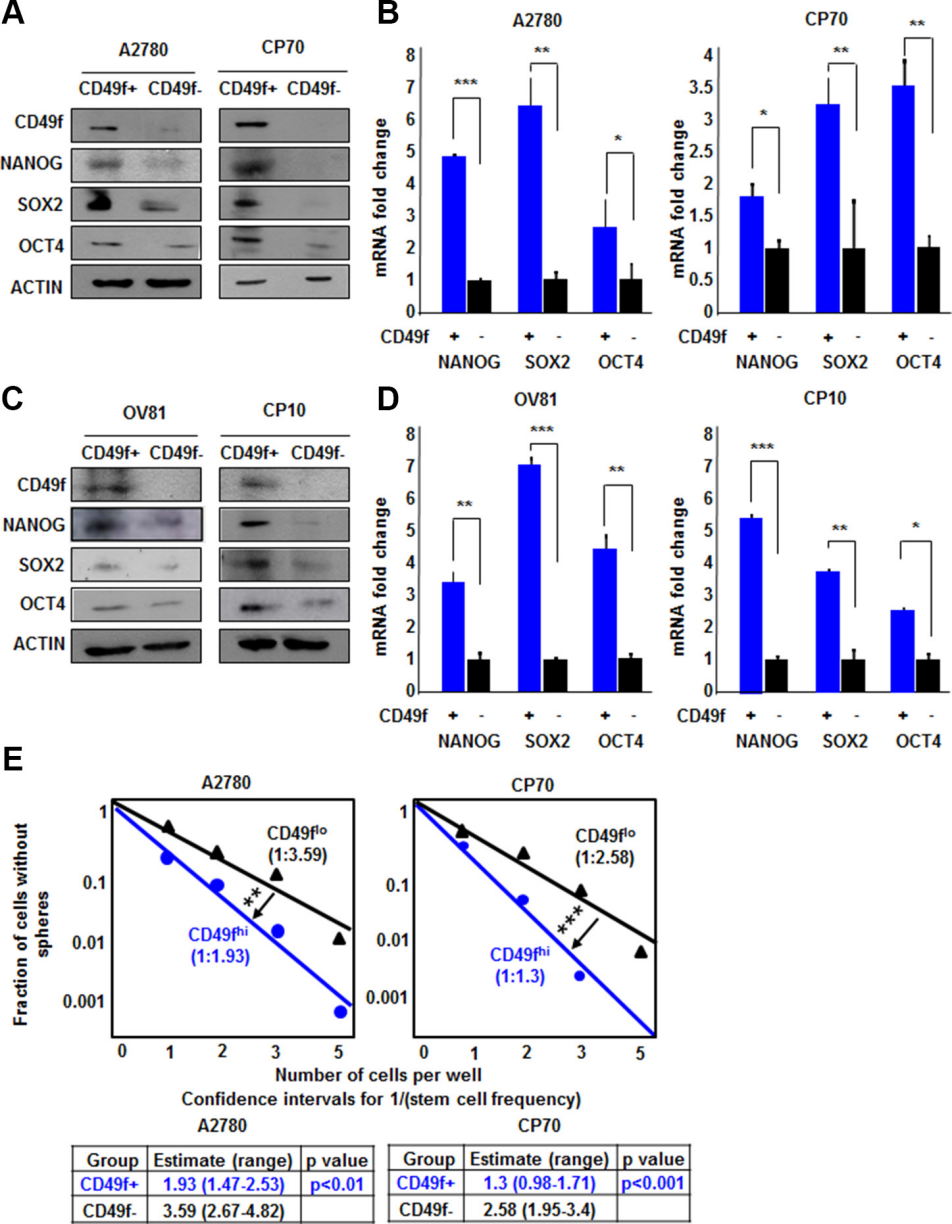
CD49f enriches CSCs in both A2780/CP70 and OV81/CP10 cells CD49f+ A2780 and CP70 cells had higher expression of NANOG, SOX2, and OCT4 proteins (**A**) and RNAs (**B**) as compared to their CD49f–counterparts. (**C**) CD49f+ OV81 and CP10 cells had higher levels of NANOG, SOX2, and OCT4 proteins as compared to their CD49f–counterparts. (**D**) Quantitation of NANOG, SOX2, and OCT4 mRNAs in CD49f-sorted A2780 and OV81 cells showed significantly higher expression levels in CD49f+ cells compared to their CD49f–counterparts. (**E**) Limiting dilution assays were performed by plating cells into 96-well plates with increasing cell numbers. CD49f+ A2780 and CP70 cells had significantly higher self-renewal capacity and stem cell frequencies as compared to their negative counterparts. Values represent mean +/− standard deviation, *p < 0.05, **p < 0.01, ***p < 0.001, as assessed by one-way-ANOVA.

Based on the inability of NANOG-GFP reporter to enrich CSC in cisplatin-resistant cells, we evaluated other CSC enrichment markers including CD49f, which we and others have previously demonstrated to be an informative CSC marker in brain tumors and breast cancer [26–28]. CD49f+ cells from both A2780 and CP70 cell lines displayed higher expression of NANOG, SOX2, and OCT4 protein and mRNA (Figure 3A, 3B). CD49f+ A2780 cells had 4.8, 6.3, and 2.5 fold higher levels of NANOG, SOX2, and OCT4 mRNA as compared to CD49f- cells. Additionally, CD49f+ CP70 cells had 1.8, 3.2, and 3.5 fold higher levels of NANOG, SOX2 and OCT4 mRNA as compared to CD49f- cells, respectively (Figure 3B). Similarly, CD49f+ cells from both OV81 and CP10 cell lines displayed higher expression of core pluripotency transcription factors (Figure 3C, 3D). In addition, CD49f enriched cancer cells with self-renewing capacity in both A2780 and CP70 cells as indicated by the difference in stem cell frequencies using the limiting dilution sphere formation assay (Figure 3E). In A2780, stem cell frequencies were 1:1.93 [confidence interval = 1:1.47–1:2.53], and 1:3.59 [confidence interval = 1:2.67–1:4.82] in CD49f+ vs CD49f- cells, respectively. In CP70, stem cell frequencies were 1:1.3 [confidence interval = 1:0.98–1:1.71], and 1:2.58 [confidence interval = 1:1.95–1:3.4] in CD49f+ vs CD49f- cells, respectively (Figure 3E). We also showed that CD49f+ cells had higher self-renewal capacity in patient-derived OV81 and CP10 cells (Supplementary Figure 4). These data support the presence of a self-renewing population in cisplatin-resistant cells that can be enriched based on CD49f.

### NANOG-GFP cells possess higher *in vivo* tumor initiation potential

**Figure 4:**
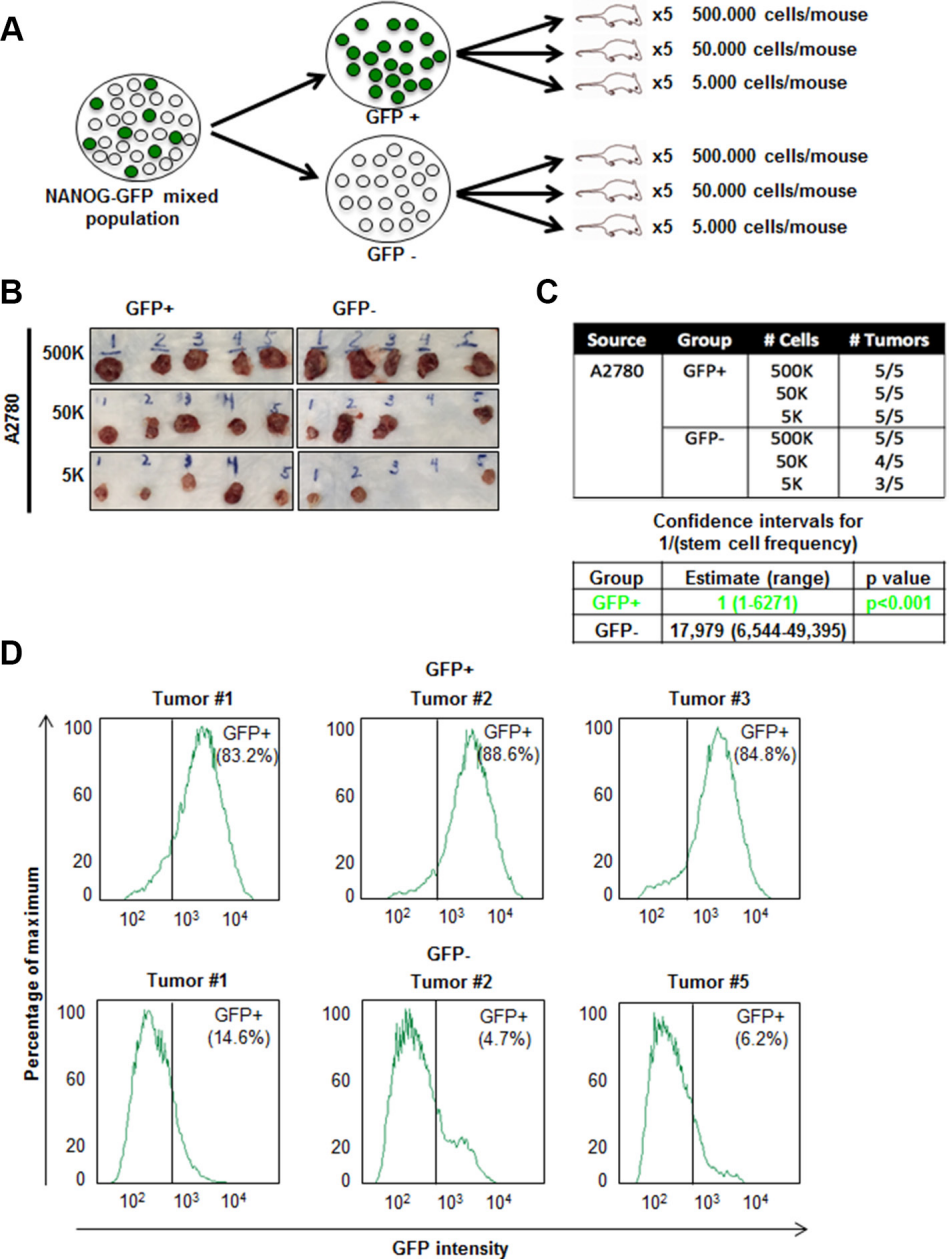
Increased *in vivo* tumor initiation frequency of A2780 GFP+ cells (**A**) Schematic of workflow of fluorescence-activated cell sorting (FACS)-sorting and injection of GFP+ and GFP–cells into NSG mice. (**B**–**C**) *In vivo* limiting dilution assay showing increased tumor initiation by GFP+ cells as compared to GFP- cells. (**D**) Cells were dissociated from excised tumors in GFP+ and GFP–groups at the end of 4 weeks. All tumors developed from initial GFP+ injections contained a population of GFP- cells and tumors originated from GFP–injections contained a population of GFP+ cells.

The gold standard functional CSC assay is tumor initiation and we wanted to assess if our reporter system could delineate difference in tumor initiation in a cisplatin-naïve context. GFP+ and GFP- populations were isolated via flow cytometry (Supplementary Figure 5A) and implanted subcutaneously into immune-compromised mice at limiting dilutions of 5,000; 50,000; and 500,000 cells to assess tumor initiation (Figure 4A). We found that GFP+ cells formed significantly more tumors than GFP- cells and had an elevated tumor initiation frequency (Figure 4B, 4C). All mice injected with GFP+ cells developed tumors whereas in mice injected with 50,000 and 5,000 GFP- cells, 4/5 and 3/5 developed tumors, respectively (stem cell frequencies were 1:1 [confidence interval = 1:6,271–1:1], and 1:17,979 [confidence interval = 1:49,395–1:6,544] in GFP+ vs GFP- cells, respectively) (Figure 4C). In addition, the tumors that formed from the initial GFP- cell injections contained GFP+ cells (ranging from 4.8–14.6%) (Figure 4D). Similarly, all 5 tumors excised from mice initially injected with GFP+ cells contained GFP- cells (Figure 4D and Supplementary Figure 5B). These data provide functional evidence that our GFP-reporter system can distinguish difference in tumor initiation potential.

### Cisplatin treatment induces stemness

**Figure 5:**
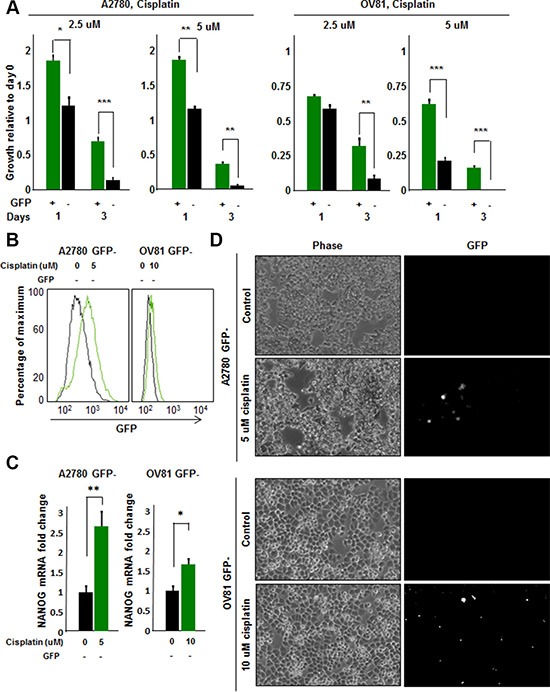
Cisplatin induces stemness in cisplatin-naïve A2780 GFP- cells (**A**) Proliferation/survival assay revealed a significant increase in growth and survival of GFP+ A2780 and OV81 cells as compared to GFP–cells at 2.5 and 5 uM concentrations of cisplatin. (**B**) Histogram of A2780 and OV81 GFP–cells treated with cisplatin. At day 3 of 5 and 10 uM cisplatin treatments, there was an increase in GFP signal as compared to untreated controls in A2780 and OV81 GFP- cells, respectively. (**C**) Quantitation of NANOG mRNA expression in GFP–A2780 and OV81 cells treated with 5 and 10 uM cisplatin, respectively. After 24 hours of cisplatin treatment, there was a dose-related increase in NANOG mRNA level in treated groups as compared to untreated control. (**D**) Photomicrographs of control and treated GFP–cells at day 3 of cisplatin treatment. Values represent mean +/− standard deviation, *p < 0.05, **p < 0.01, ***p < 0.001, as assessed by one-way-ANOVA.

**Figure 6:**
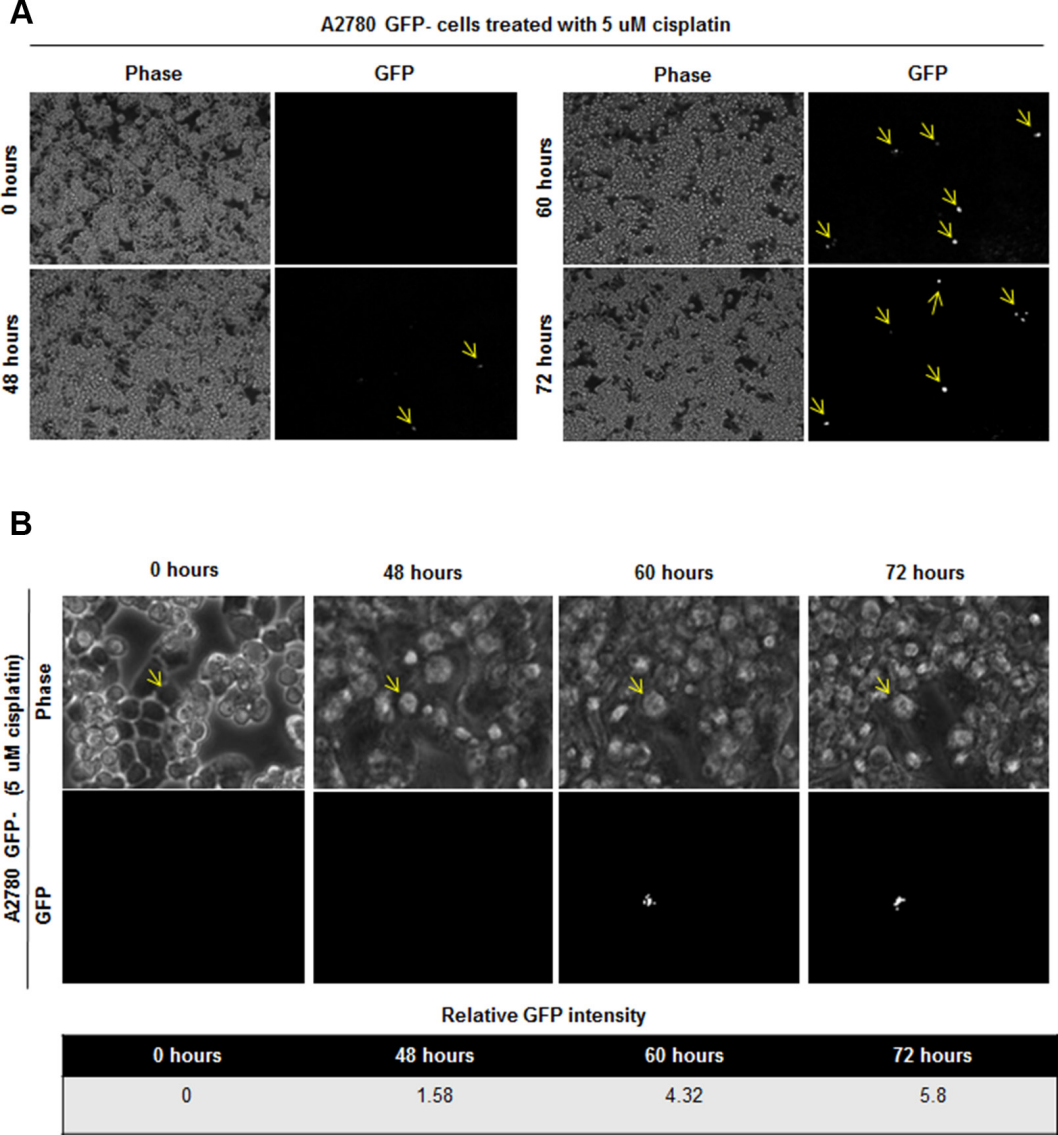
Induction of GFP signal upon 5 uM cisplatin treatment in GFP- cells (**A**) Time lapse imaging of GFP- cells treated with 5 uM cisplatin showed induction of GFP signal in initially GFP–cells. 0.6%, 0.8%, and 1.1% of cells became GFP+ at 48, 60, and 72 hours, respectively. (**B**) Tracing of one cell which is induced to become GFP+ is shown. Cell of interest indicated with yellow arrow.

Based on the presence of GFP+ cells in tumor initiated from GFP- cells, we hypothesized that the stem cell state could be activated *in vivo* and detected by our reporter system. We were particularly interested in this capacity in the context of cisplatin treatment as we observed that cisplatin-resistant cells had higher CSC activity as compared to cisplatin-naïve cells. To assess if there was a difference in cisplatin sensitivity between GFP+ and GFP- A2780 and OV81 cells, we added cisplatin to sorted GFP+ and GFP- cultures and observed a significant survival advantage in GFP+ over GFP- cells (Figure 5A). To directly interrogate the potential for cisplatin to induce a CSC state, we evaluated the response of GFP- cells to cisplatin. Upon cisplatin treatment, the surviving cells showed higher GFP intensity as compared to untreated cells (Figure 5B). We confirmed the induction of NANOG in the GFP- cells treated with cisplatin by qPCR analysis and found a significant increase in NANOG expression (Figure 5C). Namely, A2780 GFP- cells treated with 5 μM cisplatin had 2.6 fold higher levels of NANOG mRNA as compared to untreated control (Figure 5C). Similarly, GFP- OV81 cells treated with 10 μM cisplatin had 1.7 fold higher NANOG mRNA expression as compared to untreated control (Figure 5C). We observed higher GFP signal in GFP- A2780 and OV81 cells treated with cisplatin for 3 days as compared to control cells (Figure 5D). To visualize this induction, we performed time-lapse microscopy on isolated GFP- cells and found that treatment of cisplatin induced GFP expression (Figure 6A). 0.6%, 0.8%, and 1.1% of cells became GFP+ at 48, 60, and 72 hours, respectively (Figure 6A). Additionally, the raw GFP signal intensity was increased from 0 to 5.8 and 7.3 in two separate cells, respectively at day 3 (measured by the raw intensity difference of cell and the background and adjusted to cell size) (Figure 6B, Supplementary Figure 6A). As a control, we did not observe a signal in GFP channel in A2780 parental cells without NANOG-GFP promoter during 3 days of 5 uM cisplatin treatment (Supplementary Figure 6B). These data indicate that that our reporter system has the capacity to visualize dynamic changes in the stem cell state and that cisplatin treatment induces stemness.

## DISCUSSION

The CSC hypothesis is predicated on the ability to distinguish functionally distinct populations of tumor cells from patient-derived tissue, or in some cases, established cell lines. This has generally been achieved using a cell surface marker enrichment approaches. However, this approach is limited by patient-to-patient heterogeneity and caveats with cell surface marker selection, including the fluctuation of marker expression at points in the cell cycle. An alternative approach has been the detection of enzymatic activity and the most consistent marker across multiple tumors is the activity of ALDH, measured by the ALDEFLOUR assay [29]. This approach also has variability and limited by the caveats described above. Therefore, any selection approach requires functional assay validation, which are most appropriate using self-renewal sphere formation assays and/or *in vivo* tumor initiation assessments. While these methods are able to provide distinct populations for comparative analysis, the CSC state is dynamic and amenable to rapid transitions. Moreover, these approaches are limited in their ability to appreciate changes in the stem cell state in real time.

We and others have successfully demonstrated that pluripotent stem cell transcription factors, which are highly expressed in CSCs, can be leveraged to generate reporter systems that allow the interrogation of the CSC state in real time [20, 21, 25]. In triple negative breast cancer (TNBC), a tumor with a previously described CSC population [30], we have demonstrated the enrichment of a CSC population, both *in vitro* and *in vivo*, using a NANOG-promoter driven GFP reporter system [25]. This approach has allowed us to identify a novel key molecule for CSC maintenance [25], junctional adhesion molecule-A, which is also essential in brain tumor stem cell maintenance [31, 32]. Similar approaches have been developed for TNBC using elements of the SOX2 and OCT4 promoter elements (SORE6) as well as NOTCH activity [22, 24]. Based upon our success in TNBC, we applied the NANOG-GFP system to ovarian cancer and found its ability to enrich for CSCs in cisplatin-naïve cells. This approach was limited in its ability to enrich for CSCs in a population of cisplatin-resistant cells, which we found had a higher baseline of CSC transcription factor expression and self-renewal capacity (Supplementary Figure 1). In a cisplatin-resistant context, we found that CD49f was effective in distinguishing populations of cells with difference in CSC transcription factor expression and self-renewal capacity (Figure 3). These differences may be due to cisplatin driving an increase in the CSC state that is correlated with CD49f expression. Therefore, the utility of our reporter system is high in the cisplatin-naïve state, allowing us to interrogate mechanisms responsible for CSC maintenance and induction of the stem cell state.

Our results suggest that cisplatin may serve as an inductive pressure for the stem cell state. In previous studies, conventional therapies and key tumor microenvironment components have generated a stem cell selection pressure on tumor cells, resulting in the enrichment of CSCs [33, 34]. Here we have observed the direct induction of CSCs from cisplatin-naïve tumor cells *in vitro* after cisplatin treatment (Figure 6). Moreover, we also observed GFP+ cells emerging *in vivo* from GFP- cell transplantation (Figure 4), suggesting that the complex *in vivo* environment can endow the stem cell state in cells low in CSC activity. Additionally, > 80% of tumor cells excised from mice originally injected with GFP+ cells remained GFP+. One explanation might be that CSCs might have a survival advantage and selected *in vivo*. This also follows the trend that we observed in TNBC [25]. We did observe that CSCs gave rise to non-CSCs *in vivo* and the differences in GFP signal *in vitro* and *in vivo* is likely to reflect a combination of growth conditions and selective pressures. The current chemotherapeutic regimens for ovarian cancer patients involve multiple cycles of platinum-based chemotherapies, which may be promoting the stem cell state in surviving tumor cells according to our findings. An additional clinical consideration could be to block such an induction, therefore attenuating recurrence. This approach is immediately applicable as CSC-directed therapies are in clinical trials for a variety of solid tumors [35–38]. Applying this knowledge to current ovarian cancer treatment paradigms, partnering cytotoxic chemotherapy with a CSC targeted therapy, is paramount.

## MATERIALS AND METHODS

### Cell culture

The isogenic ovarian cancer cell lines A2780 (cisplatin naïve) and CP70 (cisplatin resistant), as well as patient-derived HGSOC xenografts OV81 (cisplatin naïve) and CP10 (cisplatin resistant) were cultured in log-growth phase in DMEM medium supplemented with 10% heat inactivated fetal bovine serum (FBS) at 37°C in a humidified atmosphere (5% CO_2_) [39]. At 70–90% confluence, trypsin (0.25%)/EDTA solution was used to detach cells for passaging and further experiments until passage number 15. Cisplatin was obtained from Cleveland Clinic Hospital pharmacy and 1 mg/mL stock solutions were stored at 4°C.

### Immunoblotting

Whole cell protein extracts were obtained with lysis of cells in 20 mM Tris-HCl (pH 7.5), 150 mM NaCl, 1 mM Na_2_EDTA, 1 mM EGTA, 1% NP-40, 1% sodium pyrophosphate, 1 mM β-glycerophosphate, 1 mM sodium orthovanadate, 1 ug/mL leupeptin, 20 mM NaF and 1 mM PMSF. Protein concentrations were measured with Bradford reagent (BIO-RAD, CA). Proteins in lysates (30–50 ug of total protein) were resolved by 10% SDS-PAGE and transferred to nitrocellulose membrane. Membranes were incubated overnight at 4°C with primary antibodies against NANOG (Cell Signaling), SOX2 (Cell Signaling), OCT4 (Cell Signaling), integrin α6 (Cell Signaling), and β-actin (Santa Cruz, CA). Secondary anti-mouse or anti-rabbit IgG antibodies conjugated to horse radish peroxidase (HRP) (Thermo, Rockford, IL) were used and immunoreactive bands were visualized using the ECL plus from Pierce (Rockford, IL, USA).

### Quantitative real time PCR (qPCR)

Total RNA was extracted from GFP+ and GFP, CD49f+ and CD49f–, control and cisplatin treated cells using RNeasy kit (Qiagen). For mRNA analysis, cDNA was synthesized from 1 ug of total RNA using the Superscript III kit (Invitrogen, Grand Island, NY). SYBR Green-based real time PCR was subsequently performed in triplicate using SYBR-Green master mix (SA Biosciences) on Applied Biosystems StepOnePlus real time PCR machine (Thermo). For analysis, the threshold cycle (Ct) values for each gene were normalized to expression levels of β-actin. The primers used were:

**Table:** 

β-actin	Forward	5′-AGAAAATCTGGCACCACA CC-3′
	Reverse	5′-AGAGGCGTACAGGGATAGC A-3′
NANOG	Forward	5′-CCCAAAGGCAAACAAC CCACTTCT-3′
	Reverse	5′-AGCTGGGTGGAAGAG AACA CAGTT-3′
SOX2	Forward	5′- CACATGAAGGAGCACCCG GATTAT -3′
	Reverse	5′- GTTCATGTGCGCGTAACTGT CCAT -3′
OCT4	Forward	5′-TGAGTCAGTGAAC AGGGAATG-3′
	Reverse	5′-AATCTCCCCTTTCCAT TCGG-3′

### Flow cytometry analysis

A2780 and CP70 cells at a concentration of 1 million cells/mL were sorted on BD FACS Aria II. For NANOG-GFP sorting, GFP low and high populations were sorted from NANOG-GFP promoter transduced stable cell lines. The antibodies used for FACS analysis were: APC-conjugated integrin α6 (1:100, BD Biosciences), PE-conjugated CD44 (1:100, BD Biosciences), APC-conjugated CD133 (1:100, BD Biosciences), APC-conjugated CD24 (1:100, BD Biosciences), and APC-conjugated CD117 (1:100, BD Biosciences). Appropriate isotype controls were used to set gates. For cisplatin experiments, viable cells were determined as annexin V/7-AAD negative population. Data analysis was performed on FlowJo software (Tree Star, Inc).

### Limiting dilution assays

For tumorsphere formation assays, BD FACS Aria II sorter was used to sort cells in duplicate rows of serial dilutions into 96-well ultra low attachment plates (Corning, Tewkesbury, MA, USA) with 200 uL serum-free DMEM/F12 medium per well supplemented with 20 ng/mL basic fibroblast growth factor (Invitrogen), 10 ng/mL epidermal growth factor (Biosource, Grand Island, NY, USA), 2% B27 (Invitrogen), 10 ug/mL insulin, and 1 ug/mL hydrochloride (Sigma). Tumorspheres were counted in 2 weeks under a phase contrasted microscope and data was analyzed by Extreme Limited Dilution Analysis (ELDA) platform to determine stem cell frequency (http://bioinf.wehi.edu.au/software/elda/).

### *In vivo* tumor formation

All mouse procedures were performed under adherence to protocols approved by the Institute Animal Care and Use Committee at the Lerner Research Institute, Cleveland Clinic. NOD severe combined immunodeficient (SCID) gamma (NSG) mice were purchased from the Biological Response Unit (BRU) at the Cleveland Clinic and maintained in microisolator units with free access to water and food. A2780 NANOG-GFP cells were flow sorted for GFP+ and GFP–cells and transduced with luciferase lentiviral vector construct. GFP+ and GFP–cells were then subcutaneously transplanted in three groups of serial dilutions of 5000, 50000, and 500000 cells (5 mice per group) into the right subcutaneous flank of female mice at 6 weeks of age. At 4 weeks following the injection, mice were euthanized and the tumors were resected.

### Cell proliferation/survival assay

A2780 NANOG-GFP cells were sorted for GFP+ and GFP- cells and plated in 6-well plates at 200,000 cells/well and treated on the next day with cisplatin at the doses of 2.5 and 5 uM doses. The number of live cells in control and treatment groups were manually counted using hemacytometer at days 1, 3, and 5 using Trypan blue dye exclusion as a live cell marker. Fold changes in growth relative to day 0 were assessed. The experiment was repeated with CD49f+ and CD49f- cells.

### Time lapse imaging

A2780 NANOG-GFP cells were sorted and GFP- cells were plated in 6-well plates at 200,000 cells/well and treated with cisplatin at 2.5 and 5 uM doses. A2780 parental cells which donot harbor the reporter, and untreated A2780 GFP- cells were used as controls. Cells were monitored with LEICA DMI600 inverted microscope with environmental chamber for 3 days, and phase and GFP images were collected every 5 minutes. Induction of GFP signal was calculated based on the raw intensity difference of the cell and the background, and adjusted to cell size.

### Statistical analysis

Data are presented as mean +/− standard deviation. One-way ANOVA analysis was used to calculate statistical significance and *p* < 0.05 was considered significant.

## SUPPLEMENTARY MATERIALS FIGURES


