# Supplementary material for: Cisplatin induces stemness in ovarian cancer
Source: Oncotarget. 2016 Apr 20;7(21):30511–22. doi: 10.18632/oncotarget.8852 (PMC5058697; doi:10.18632/oncotarget.8852)
Supplement: Supplementary file 1 [file oncotarget-07-30511-s001.pdf]

Cisplatin induces stemness in ovarian cancer

Supplementary Materials

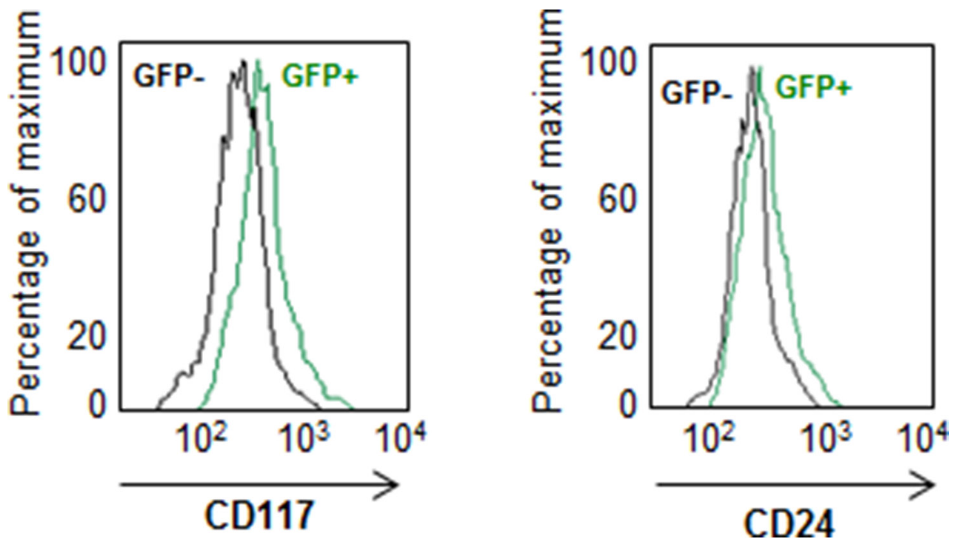

Supplementary Figure S1: A2780 GFP+ cells have higher expression of CD117 and CD24. A2780 NANOG-GFP cells were stained with APC-CD117 and APC-CD24, and analyzed by flow cytometry. The green and black dots and histogram lines represent GFP+ and GFP− cells, respectively. Histograms demonstrate that GFP+ cells are enriched in CD117 and CD24 expressions.

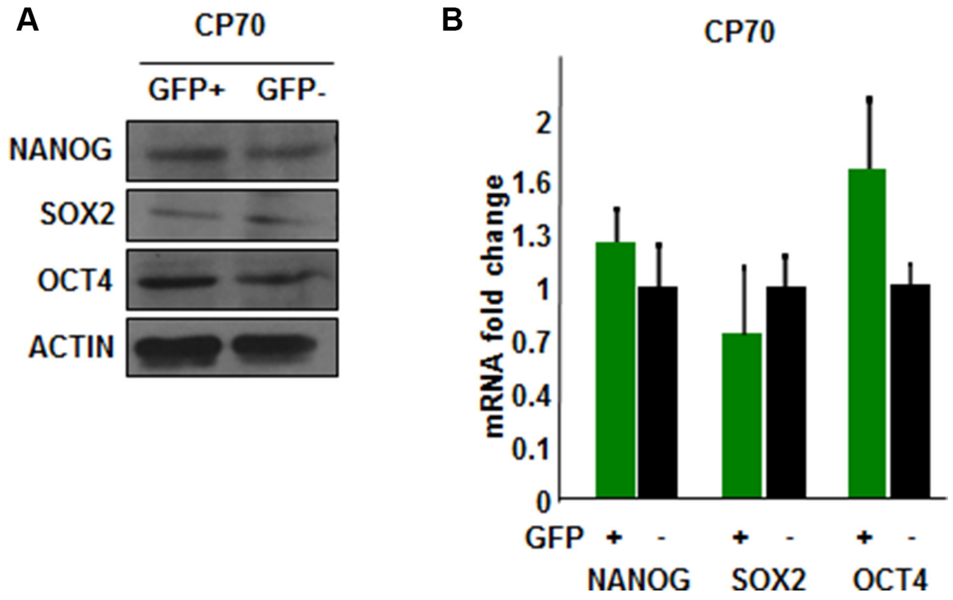

Supplementary Figure S2: NANOG-GFP reporter does not enrich CSCs in CP70 cells. (**A**) Immunoblots of GFP-sorted CP70 cells probed with antibodies to stem cell transcription factors demonstrate no significant difference between GFP+ and GFP− CP70 cells. Actin was used as a loading control. (**B**) Similarly, quantitation of mRNA levels does not show a difference between GFP+ and GFP− populations.

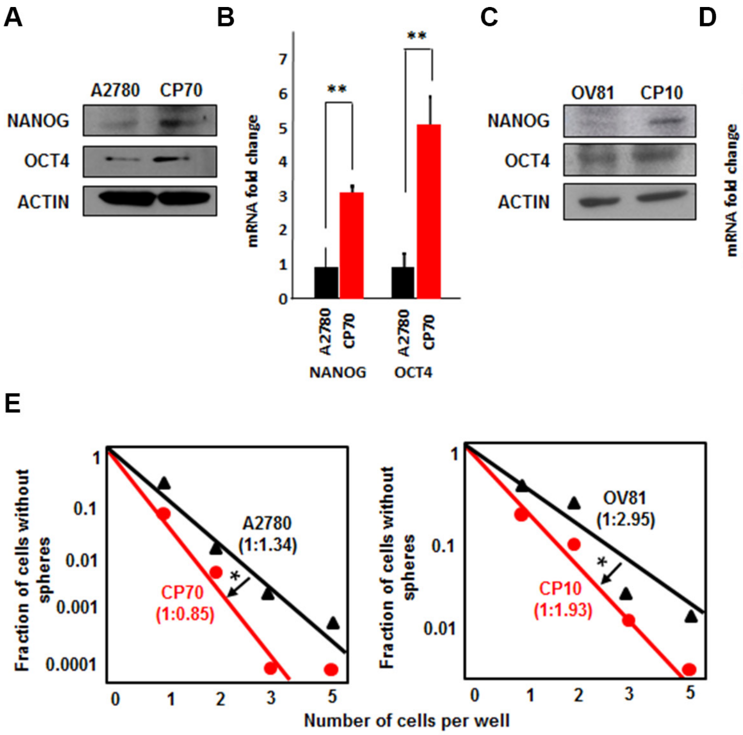

Confidence intervals for 1/(stem cell frequency)

Group	Estimate (range)	p value
CP70	0.85 (0.62-1.16)	p<0.05
A2780	1.34 (1.013-1.76)	

Group	Estimate (range)	p value
CP10	1.93 (1.47-2.52)	p<0.05
OV81	2.95 (2.22-3.92)	

Supplementary Figure S3: CP70 cells have higher expression of CSC markers and self-renewal. (**A**) Immunoblots of A2780 and CP70 parental cells demonstrate higher levels of NANOG and OCT4 transcription factors in CP70 cells. Actin was used as a loading control. (**B**) Quantitation of NANOG and OCT4 mRNA expressions in A2780 vs CP70 cells showed higher levels of these transcripts in CP70 cells. (**C**) CP10 cells have higher expression of NANOG and OCT4 proteins as compared to OV81 cells. (**D**) Quantitation of NANOG and OCT4 mRNA expressions in OV81 vs CP10 cells showed higher levels of these transcripts in CP10 cells. (**E**) CP70 and CP10 parental cells had higher self-renewal capacity and stem cell frequency as compared to A2780 and OV81 parental cells, respectively. Values represent mean +/– standard deviation, *$p < 0.05$, **$p < 0.01$, as assessed by one-way-ANOVA.

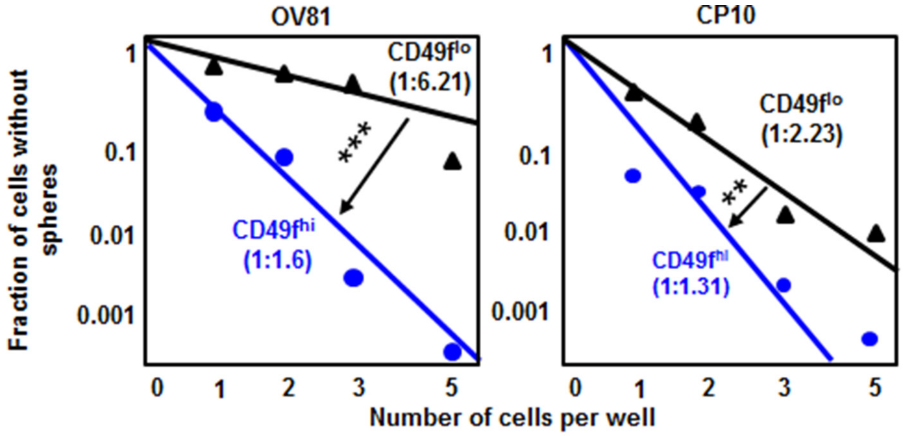

Confidence intervals for 1/(stem cell frequency)

OV81

Group	Estimate (range)	p value
CD49f+	1.6 (1.22 – 2.1)	<0.001
CD49f-	6.21 (4.39 – 8.8)	

CP10

Group	Estimate (range)	p value
CD49f+	1.31 (1 – 1.74)	0.006
CD49f-	2.23 (1.69 – 2.93)	

Supplementary Figure S4: CD49f+ OV81/CP10 cells have higher self-renewal capacity. CD49f+ OV81 and CP10 cells had higher self-renewal capacity and stem cell frequency as compared to their CD49f– counterparts.

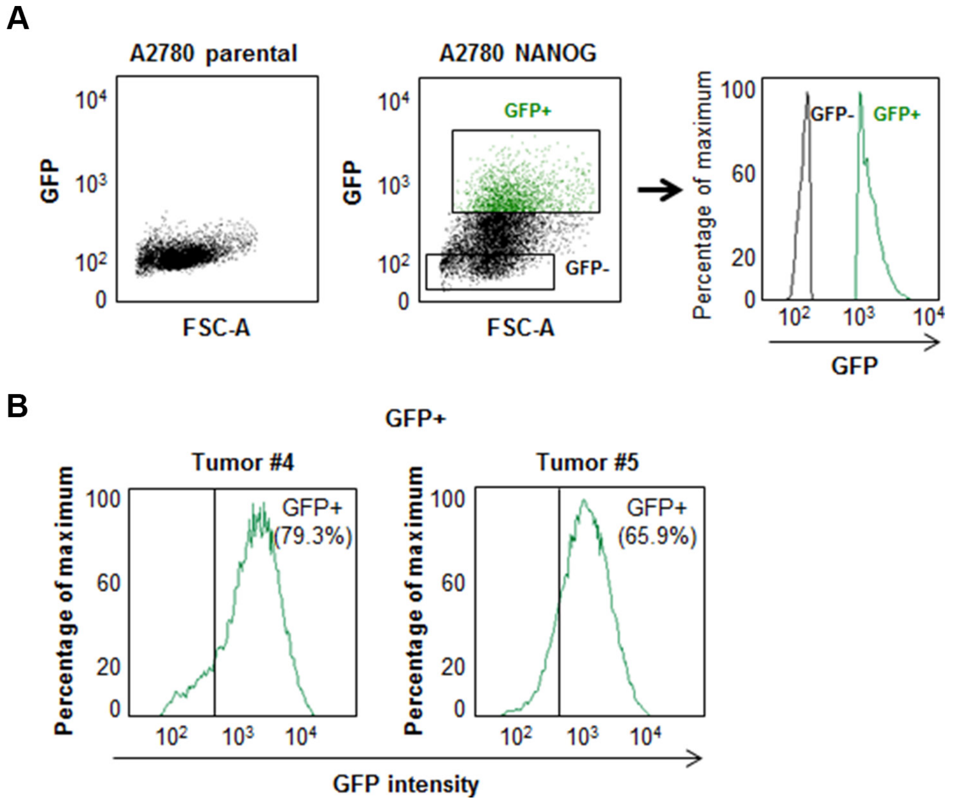

Supplementary Figure S5: Validation of FACS-sorting prior to *in vivo* injections and GFP signal intensity of additional tumors initiated from GFP+ cells. (**A**) NANOG-GFP reporter transduced cells are sorted for 20% top and bottom GFP intensity and designated as GFP+ and GFP–, respectively. These populations are subsequently injected into mice to study tumor initiation. (**B**) The additional 2 tumors developed from initial GFP+ injections also contained a population of GFP–cells.

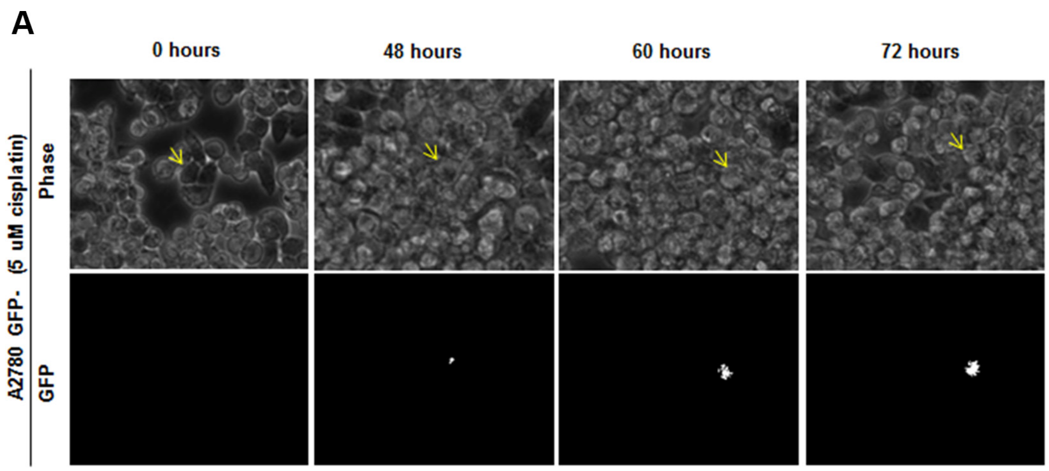

Relative GFP intensity

0 hours	48 hours	60 hours	72 hours
0	1.8	5.41	7.3

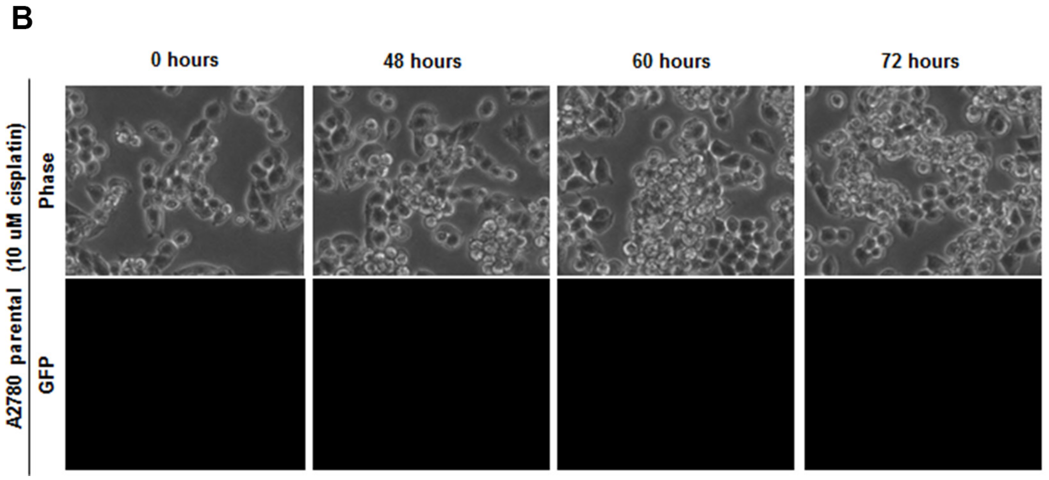

Supplementary Figure S6: Cisplatin induced GFP induction in another GFP- cell while there was no GFP signal in cells without NANOG-GFP reporter. (**A**) Induction of GFP signal upon cisplatin treatment in another GFP- cell. Time lapse imaging and tracing of one GFP- cell demonstrated induction of GFP signal at 5 uM cisplatin treatment. (**B**) Treatment of A2780 parental cells which did not contain NANOG-GFP reporter, did not give any signal at GFP channel.
